# Time to Redefine PD? Introductory Statement of the MDS Task Force on the Definition of Parkinson's Disease

**DOI:** 10.1002/mds.25844

**Published:** 2014-03-11

**Authors:** Daniela Berg, Ronald B Postuma, Bastiaan Bloem, Piu Chan, Bruno Dubois, Thomas Gasser, Christopher G Goetz, Glenda M Halliday, John Hardy, Anthony E Lang, Irene Litvan, Kenneth Marek, José Obeso, Wolfgang Oertel, C Warren Olanow, Werner Poewe, Matthew Stern, Günther Deuschl

**Affiliations:** 1 Department of Neurodegeneration, Hertie Institute for Clinical Brain Research and German Center of Neurodegenerative DiseasesTuebingen, Germany; 2 Department of Neurology, Montreal General HospitalMontreal, Quebec, Canada; 3 Department of Neurology, Radboud University Nijmegen Medical CenterNijmegen, the Netherlands; 4 Xuanwu Hospital of Capital Medical UniversityBeijing, People's Republic of China; 5 Department of neurology, Salpêtrière Hospital, APHP, University Paris 6UPMC, Paris, France; 6 Rush University Medical CenterChicago, Illinois, USA; 7 Neuroscience Research, Randwick, Australia and the University of New South WalesSydney, Australia; 8 Department of Molecular Neuroscience, UCL Institute of NeurologyLondon, UK; 9 Edmond J Safra Program in Parkinson's Disease, Division of Neurology, Toronto Western Hospital and the University of TorontoToronto, Canada; 10 Department of Neurosciences, University of California San DiegoLa Jolla, California, USA; 11 Institute for Neurodegenerative DisordersNew Haven, Connecticut, USA; 12 University of Navarra-FIMAPamplona, Spain; 13 Department of Neurology, Philipps University of MarburgMarburg, Germany; 14 Department of Neurology, The Mount Sinai HospitalNew York, New York, USA; 15 Department of Neurology, Innsbruck Medical UniversityInnsbruck, Austria; 16 Penn Neurological InstitutePhiladelphia, Pennsylvania, USA; 17 Department of Neurology, Christian-Albrechts UniversityKiel, Germany

**Keywords:** redefinition of PD, gold standard, subtypes, disease heterogeneity, nonmotor prodrome, MDS diagnostic criteria

## Abstract

With advances in knowledge disease, boundaries may change. Occasionally, these changes are of such a magnitude that they require redefinition of the disease. In recognition of the profound changes in our understanding of Parkinson's disease (PD), the International Parkinson and Movement Disorders Society (MDS) commissioned a task force to consider a redefinition of PD. This review is a discussion article, intended as the introductory statement of the task force. Several critical issues were identified that challenge current PD definitions. First, new findings challenge the central role of the classical pathologic criteria as the arbiter of diagnosis, notably genetic cases without synuclein deposition, the high prevalence of incidental Lewy body (LB) deposition, and the nonmotor prodrome of PD. It remains unclear, however, whether these challenges merit a change in the pathologic gold standard, especially considering the limitations of alternate gold standards. Second, the increasing recognition of dementia in PD challenges the distinction between diffuse LB disease and PD. Consideration might be given to removing dementia as an exclusion criterion for PD diagnosis. Third, there is increasing recognition of disease heterogeneity, suggesting that PD subtypes should be formally identified; however, current subtype classifications may not be sufficiently robust to warrant formal delineation. Fourth, the recognition of a nonmotor prodrome of PD requires that new diagnostic criteria for early-stage and prodromal PD should be created; here, essential features of these criteria are proposed. Finally, there is a need to create new MDS diagnostic criteria that take these changes in disease definition into consideration. © 2014 The Authors. *Movement* Disorders published by Wiley Periodicals, Inc. on behalf of International Parkinson and Movement Disorder Society.

Since the original description of Parkinson's disease (PD), there have been radical changes in our conceptualization of the disease, starting with a better understanding of motor manifestations, clear pathologic definitions, and availability of therapy that is so reliably effective as to be part of diagnostic criteria. Moreover, our understanding continues to develop, with increasing knowledge of nonmotor aspects, recognition that neurodegeneration can start before motor symptoms manifest, better understanding of genetics and environmental factors, and progress toward developing reliable biomarkers and animal models. With these advances, clinicians and specialists in genetics, epidemiology, pathology, and basic science have developed their own conceptualizations of disease; each valid, but none representing the whole truth. Can these conceptualizations be united under one umbrella definition?

To deal with the challenges of disease definition, the International Parkinson and Movement Disorder Society (MDS) convened a task force to generate an updated definition of PD. The group identified three critical issues: (1) Who decides what is PD and what is the gold standard for a “final” diagnosis? (2) What clinical features fit under the PD umbrella? Should subtypes be incorporated into the diagnosis? Should some diagnostic entities be held distinct (e.g., dementia with Lewy bodies; DLB)? (3) Defining disease onset—can PD be defined before classic motor features develop?

This review is intended as an introductory discussion article; it is not the final word on disease definition, but rather an opening of dialog. Each section will start by presenting conversational-style informal minivignettes (in italics) that summarize what clinicians or researchers often mention when pointing out problems with the current PD definition. Both sides of each issue are then discussed, followed by proposals for moving forward. Finally, we will discuss the need for new diagnostic criteria for PD.

## What Is the Gold Standard for the Definition of PD?

The issue: *A patient with classic clinical PD died without autopsy; can one never say they are “sure” she had PD? Why is autopsy the gold standard if it is almost never available (and might become outdated, once we have good biomarkers). Furthermore, don't genetic studies suggest that pathology can be inconclusive? A patient from a family of pathologically confirmed LRRK2 PD who meets clinical criteria for PD, but has no Lewy bodies (LB) on autopsy, or patients with parkin mutations without LB; do they not have PD?*

What we currently consider as the gold standard for PD diagnosis is not always explicit. Nevertheless, most clinicians would endorse the diagnostic gold standard as a combined clinical and pathological syndrome, consisting of the following:

1. A motor clinical syndrome, with levodopa-responsive parkinsonism, typical clinical characteristics, and an absence of markers suggestive of other disease.
2. Pathologic confirmation of α-synuclein (α-Syn) deposition and dopamine neuronal loss in the substantia nigra pars compacta (SNpc). Only at this point is the diagnosis termed “definite.” If typical synuclein pathology is not found, the clinical diagnosis is considered incorrect. Likewise, the pathology is “incidental” in the absence of clinical symptoms or attributed to another disease if parkinsonism did not dominate the clinical picture (e.g., DLB or primary autonomic failure).

Therefore, a motor clinical syndrome is the entry point, and pathology is the arbiter of diagnosis. Pathologic findings ultimately feed back into clinical criteria by correcting clinical diagnoses.

### Challenging the Status Quo

Several challenges have been raised against the requirement for both SNpc neurodegeneration and α-Syn deposition as essential features and, more globally, of pathology's role as the ultimate arbitrator. These include the following:

1. Genetic/pathologic correlation: Although, in many patients, leucine-rich repeat kinase 2 (LRRK2) mutations are associated with classic α-Syn deposition,^1^,^2^ there are nonetheless reports of members of the same family having different pathologies, some without α-Syn deposition. LB are absent in 21% of G2019 mutations and >50% of non-G2019S mutations.^2^ With *parkin*, most meet clinical PD criteria, have SN degeneration on autopsy, but have little or no α-Syn deposition^2^ (exceptions were older and may have had coincidental “incidental LB”). We must also take into account the uncertain role of α-Syn aggregation (possibly protective) versus α-Syn oligomers (toxic) in PD pathogenesis. As a hypothetical illustration, imagine a genetic mutation that prevents aggregation of “toxic” α-Syn oligomers into “protective” LB; this mutation would recapitulate the PD pathogenic process, without α-synuclein deposition on autopsy.
2. SNpc loss is not the only pathologic feature and may not occur first: The premotor/prodromal syndrome must also have a pathologic correlate (see “The Beginning of PD” below).
3. α-Syn pathology may not determine clinical symptoms: On autopsy, approximately 15% have incidental LB, 5 times the prevalence of clinical LB diseases.^3^–^6^ If α-Syn pathology defines disease alone, must all these patients be considered asymptomatic PD?
4. α-Syn aggregates may not be the first pathology: Lewy pathology may develop relatively late, with presynaptic dysfunction being the first abnormality.^7^–^9^ Might current pathologic criteria miss early stages of PD?
5. Pragmatic considerations: Autopsy is performed only at the end of life (not useful diagnostically during life), and only few have pathologic confirmation.

Potential alternate gold standards for diagnosis could include the following:

1. Clinical diagnosis alone: James Parkinson did not define a pathologic diagnosis, but a clinical syndrome. This clinical syndrome is readily recognized and, despite interindividual variability, very characteristic. Patients experience clinical disease, not pathology, and clinical criteria contain the features important to patients, including response to treatment.
2. Genetics: If experience with *parkin-* and LRRK2-related parkinsonism suggests that pathology can be inconclusive, can genetics ultimately define diagnosis?

### Defending the Status Quo

Despite these issues, are there reasons to keep our current clinicopathologic standard?

1. Alternate gold standards also have limitations:
  - Clinical: Defining PD solely on a clinical basis is circular; you can never be wrong. If clinical diagnosis is the gold standard, a patient who meets clinical criteria has PD regardless of autopsy findings. What of patients with clinical PD diagnosis who have clear pathology of alternate diagnoses, such as MSA or PSP?
  - Genetics: Well under 10% of PD patients currently have a genetic explanation. Moreover, whereas new genetic causes may be defined, heritability is relatively low; most monozygotic twins are discordant.^10^,^11^ Also, if genes are inpenetrant, interpreting a positive genetic test as diagnostic can be incorrect. Finally, genetic mutations may result in heterogeneous clinical phenotypes, not just heterogeneous pathology; for example, LRRK2 mutations are also associated with motor neuron disease.^12^ If a LRRK2 carrier had classic clinical motor neuron disease, would genetic criteria mandate diagnosis of PD?
2. Although pragmatic considerations imply that pathological diagnosis is not useful for an individual, pathology can nonetheless serve as an ultimate reference by which clinical criteria are tested. Furthermore, if biomarkers of both α-Syn deposition and SNpc degeneration become sufficiently reliable, these could ultimately replace autopsy confirmation.
3. *Parkin* and similar mutations might have a different underlying pathogenesis than sporadic PD. Moreover, these patients often have important clinical differences (e.g., earlier age of onset). It thus might be entirely appropriate to consider them separate diseases or a subcategory of PD.
4. Finally, it should not be forgotten that the vast majority (∼95%) of patients who fulfill clinical criteria have classic α-Syn pathology. Moreover, when the clinical diagnosis is “wrong,” there is usually a clear alternate pathologic cause (PSP, MSA, and so on). If we let exceptional cases drive our definition, do we “throw out the baby with the bathwater?”

### Moving Forward

***The task force proposes*** the following:

1. The core clinicopathologic criteria of a clinical motor syndrome accompanied by SNpc neurodegeneration and synuclein deposition remain a gold standard of PD diagnosis. In the future, should reliable biomarkers of synuclein deposition be developed, these can be used to indicate a likely gold-standard clinicopathologic diagnosis.
2. To incorporate genetic findings under the PD umbrella, a separate “clinicogenetic” category should be created to diagnose PD, regardless of the occurrence of synuclein deposition. This category would refer specifically to highly penetrant mutations in which the majority of affecteds meet clinical PD criteria, regardless of whether autopsy specimens of patients with this mutation find α-Syn pathology. In research studies, this diagnostic subcategory could be included or not according to the context. For example, an autopsy study validating clinical diagnostic criteria might exclude such patients, a randomized trial of symptomatic dopaminergic therapy might include them, and a neuroprotective trial may elect to include or exclude, depending upon the mechanism of the agent.
3. A new scheme is likely needed to replace the current PARK classification, which is under considerable strain. This scheme should specifically differentiate between causative genes and risk factors, consider the predominant phenotype, and—in the long run—admit the incorporation of protective variants.^13^ A dedicated task force of the MDS is currently working on this complex issue.

## What Features Fit Under the PD Umbrella?

### DLB

The issue: A patient developed cognitive impairment 18 months after PD diagnosis; he has PD dementia (PDD). Another developed cognitive impairment 10 months after PD diagnosis; according to current definitions, the initial diagnosis was “wrong,” and she has DLB. Does this make sense?

As diagnostic criteria currently stand, dementia developing before the second year of parkinsonism is an exclusion criterion for PD; the diagnosis is DLB.^14^–^16^ If dementia starts after 1 year, the diagnosis is PDD.

### Challenging the Status Quo

Beyond the arbitrary nature of the 1-year rule, there is increasing controversy about whether the distinction itself is valid. Briefly, the challenges are^16^ as follows:

1. Similar dementia presentation, with hallucinations, fluctuations, neuroleptic sensitivity, and rapid eye movement sleep behavior disorder (RBD) in both.^16^,^17^
2. Similar neuropsychological findings, with predominant visuoperceptual impairment, improvement of memory with cueing, and so on.^16^
3. Similar nonmotor profile, with olfactory loss, depression, autonomic dysfunction, and sleep disorders in both.
4. Similar imaging, with overlapping patterns of cortical atrophy, glucose utilization, neurotransmitter changes,^18^ and diffusion tensor imaging.^19^
5. Similar prodromal stage: For example, patients with idiopathic RBD develop both syndromes, with little difference in clinical evolution patterns.^20^
6. Similar genetics: For example, family members with α-Syn duplications or triplications as well as glucocerebrosidase mutations can present with either condition.
7. Similar pathology: Patients in both groups demonstrate α-Syn pathology in both the brainstem and cortex.

Implicit in this discussion is the conceptualization of DLB, which shares clinical and pathological features of Alzheimer's disease (AD) and PDD. This overlap has created tension in naming the condition, with evolution from “Lewy body variant of AD,” to “Lewy body dementia,” to the current “dementia with Lewy bodies.”

### Defending the Status Quo

There are several reasons to consider retaining the current distinction:

1. Although not universally specific, there are imaging differences between PDD and DLB, including degree of amyloid deposition, cholinergic loss, vascular damage, single-photon emission CT/PET hypoperfusion, and atrophy patterns.^16^,^21^
2. Parkinsonism in DLB can differ from idiopathic PD, typically with less tremor and l-dopa response.^22^ Many parkinsonian DLB patients would not meet criteria for clinical PD.
3. Although patients with DLB and PDD look similar at end stage, the course can be extremely different. A counterexample to the vignette: A patient diagnosed as AD with a severe amnestic disorder eventually develops visual hallucinations, cognitive fluctuations, and mild parkinsonian signs (DLB). Another patient has typical PD with normal cognition for many years and eventually develops hallucinations and cognitive decline (PDD). Are these patients not distinct?

### Moving Forward

**Figure 1:**
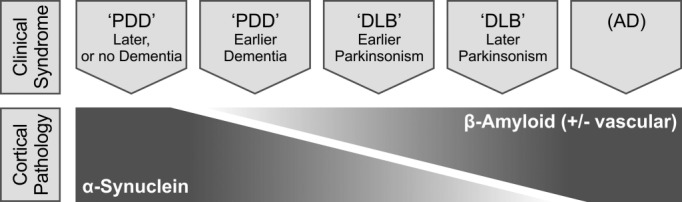
Simplified model of how the type of cortical pathology and clinical presentation of PDD and DLB might interact. Dementia in DLB/PD is associated with two major pathologies: synucleinopathy (i.e., Parkinson pathology) and neuritic amyloidopathy (i.e., Alzheimer pathology). In PD patients who develop dementia very late in their illness, or not at all (far left), neuritic amyloid deposition is minimal (or absent), and cortical pathology is mainly that of α-Syn deposition. At the other extreme, DLB patients with predominant neuritic amyloid deposition and very minimal α-Syn deposition would usually be diagnosed as AD during life, developing clinical DLB hallmarks late (if at all). Between these two extremes of the spectrum lie the most patients with PD and DLB. This hypothetical spectrum is not meant to be exclusive—other factors may also be important in determining dementia onset (e.g., “top down” vs. “bottom up” α-Syn deposition, degree of vascular pathology, and so on).

Despite the controversy, most agree that there is important clinicopathologic overlap between these conditions. Perhaps in DLB and PDD, there is a continuum between relatively “pure” α-Syn pathology and a predominant Alzheimer (± vascular) pathology (Fig. 1). Most DLB patients will develop parkinsonism and most PD patients will develop dementia, but onset time differs. At one extreme are patients with young-onset classic PD who remain dementia free for most of their long disease course; if they eventually dement, cortical pathology would be predominantly LB, with sparse AD pathology. At the other extreme are patients who first develop dementia and manifest parkinsonism very late or never during life; these patients would have more AD pathology (± vascular pathology) with less LB deposition. Between these extremes lie the majority of patients. If a continuous spectrum exists, the advantages of categorical division are debatable.

Therefore, ***the task force proposes*** the following:

Consider omitting the “1-year” rule, which separates PDD and DLB. Rather, when a patient presents with motor signs *and* meets full clinical criteria for PD, the diagnosis of PD is applied, regardless of presence or timing of dementia. In other words, dementia is no longer an exclusion criterion for PD. For those patients who already carry a DLB diagnosis (according to consensus criteria^23^), the diagnosis can optionally be qualified as “PD (DLB subtype).”

Note that this proposal would not invalidate the diagnostic category of DLB. In clinical communication with patients with the DLB subtype, the diagnostic term DLB could continue to be used. Moreover, the classification of patients with DLB diagnosis who do not have parkinsonism, or whose parkinsonism does not not meet PD criteria (e.g., no response to dopaminergic medications), would not be affected by this definition change.

## PD Subtypes

The issue: A patient had unilateral tremor onset at age 40, robust l-dopa response with fluctuations and, 20 years later, has few nonmotor features. A second developed bilateral bradykinesia and rigidity at age 80 and had no fluctuations, but had severe constipation, urinary dysfunction, sleep disturbance, and depression, eventually dying with dementia. Do these patients have the same disease?

### Challenging the Status Quo

PD has considerable variability in rate of progression of motor manifestations and prevalence of nonmotor manifestations. This can question the conceptualization of PD as a unitary disorder. At minimum, it suggests that PD may be divisible into subtypes. Currently, no formal subtype classification exists and subtypes have not been generally incorporated into research protocols.^24^

Most attempts to classify subtypes rely upon one of two approaches. One uses data-driven approaches with cluster analysis to identify subtypes in a hypothesis-free manner.^25^–^32^ In these studies, clusters have been defined based on age, motor features, and, possibly, a nonmotor symptom cluster.^26^ These variables correlate with different mortality, progression, motor complications, dementia, and behavioral symptoms. The second approach classifies subgroups on a single factor and looks for differences between groups. Two commonly suggested subtypes are onset age and tremor dominance. Age subtyping is proposed because young patients have more robust l-dopa response, more motor complications, and fewer cognitive disturbances.^33^–^35^ Tremor-predominant subtypes are proposed because they may have better prognosis and fewer cognitive disturbances.^33^–^35^ Other studies suggest other potential subtype identifiers; for example, olfactory loss correlates with cognitive impairment,^36^ and RBD correlates with autonomic dysfunction and higher dementia risk.^37^,^38^ In the future, it may also be possible to subdivide PD according to pathogenic mechanism (mitochondrial predominant, inflammatory predominant, and so on).

### Defending the Status Quo

1. Regardless of the dramatic differences in clinical manifestations, most patients with PD have a characteristic pathologic substrate, suggesting that a unifying diagnosis is appropriate. Moreover, many other diseases have dramatic differences in clinical severity and manifestations.
2. Current subtypes may not impact much upon disease course. Some studies find that tremor has little effect on prognosis.^32^,^34^ Where there are differences, there are potential confounds, including diagnostic sensitivity (tremor-dominant patients are diagnosed earlier, so prognosis appears better for disease duration), or misdiagnosis (patients misdiagnosed as tremor-predominant PD have relatively benign alternate diagnoses, such as essential tremor, versus akinetic-rigid syndrome alternate diagnoses, such as MSA or PSP). Age is continuous, and defining robust cutoffs is not simple. For example, young cutoffs (e.g., <40) help identify genetic mutations, but represent only a small proportion of PD patients (<1%).^39^,^40^ Criteria for “old-onset” PD may also be needed, because older patients have worse prognosis, higher mortality, more dementia, and more mixed pathology. Finally, current age, rather than onset age, may be the critical factor influencing disease manifestations.

### Moving Forward

In summary, there is no doubt that PD has considerable heterogeneity in clinical presentation and prognosis. ***The task force proposes*** the following:

1. Clinical subtypes should only be delineated if there are clear data that demonstrate consistent, large differences in prognosis, predicted disease manifestations, or treatment. Currently, it is unclear whether any of the current subtype classifications qualify.
2. The search for subtypes should not be restricted to clinical features, but should include subtypes of molecular pathogenesis.

## The Beginning of PD

The issue: A patient has RBD, olfactory loss, constipation, and depression, but no parkinsonism. Dopaminergic neuroimaging and SN ultrasound are abnormal. Doesn't this patient have PD?

### Challenging the Status Quo

It has become clear that neurodegeneration in PD likely begins years or decades before full PD diagnosis can be made.^41^ In this phase, nonmotor manifestations, such as RBD, hyposmia, autonomic dysfunction, visual disturbances, and depression, often occur. This has important implications for defining PD. The patient described above almost certainly has neurodegeneration with α-Syn pathology and therefore, in some sense, “has” PD. If so, should our definition be changed?

### Defending the Status Quo

Before adding early stages to a formal PD definition, the following caveats should be noted:

1. Autopsy studies find incidental LB disease in 15% of the elderly, but only a minority manifest clinical symptoms. This suggests that there may be compensatory mechanisms that forestall progression, so transition to clinical PD may not be inevitable.
2. Most premotor signs are nonspecific (e.g., olfactory dysfunction,^42^ constipation,^43^ and SN hyperechogenicity^44^), reducing accuracy of early diagnosis.
3. Neurodegeneration in PD may not universally start outside the SNpc. Moreover, not all PD patients have nonmotor features, especially in early motor stages.
4. Given that PD definition has always centered on a motor syndrome, changing the definition to include patients without motor signs is a major shift. It would radically change communication with patients, estimates of disease prevalence, and so on. For continuity with historical standards, it may be preferable to retain the motor syndrome in “classic” PD diagnosis and delineate prodromal stages separately.

### Moving Forward

Regardless of the caveats listed above, there is little question that many patients have a preclinical/prodromal stage of their disease, which should be incorporated in the definition of PD.^45^,^46^ ***The task force proposes*** the following:

1. Clinical diagnosis of classic PD should remain centered on a motor syndrome. Separate research-based criteria should be developed to allow diagnosis of early PD stages.
2. For delineation of early stages, disease should be classified as “preclinical” and “prodromal.” Preclinical refers to the presence of neurodegenerative synucleinopathy without clinical symptoms (i.e., defined by biomarkers; note that this stage cannot be currently diagnosed because reliable biomarkers are not available). Prodromal refers to the presence of early symptoms and signs before the classical PD diagnosis is possible. This prodromal term makes no assumptions about the order in which motor versus nonmotor symptoms develop.
3. Because one cannot determine whether any patient with prodromal neurodegenerative synucleinopathy will eventually progress to full clinical PD, the definition of prodromal PD should center upon the likelihood of a neurodegenerative synucleinopathy being present, regardless of “conversion rate” to full clinical PD.
4. Although PD, by definition, passes inevitably through some type of prodromal phase, there are currently no 100% reliable means to identify prodromal PD. Therefore, diagnostic criteria for prodromal PD will necessarily be probabilistic. We propose two levels of certainty. Probable prodromal PD would refer to a high likelihood (e.g., >80%; sufficiently certain for neuroprotective trials). Possible prodromal PD would refer to a lower, but still substantial, likelihood of neurodegenerative synucleinopathy (e.g., 30%-80%).
5. Prodromal PD criteria should incorporate clinical motor markers, clinical nonmotor markers, and nonclinical biomarkers. Inclusion of a marker into prodromal criteria should generally require prospective studies documenting predictive value for full clinical PD. Markers should be divided into categories of specificity, such that high specificity markers carry more weight than those with lower specificity. Criteria should also incorporate risk, adjusting probability estimates for persons with documented high-risk states (e.g., carriers of genetic mutations).

## From Definition to Diagnostic Criteria

When a disease definition changes, its diagnosis inevitably changes with it. Therefore, creation of a new definition provides an opportunity to develop new clinical diagnostic criteria. There are several reasons to develop new clinical diagnostic criteria:

1. No standard clinical criteria exist. Many criteria have been proposed for PD,^45^–^52^ but none have been developed or adopted by an international organization such as the MDS.
2. New knowledge: Diagnostic criteria must accommodate new findings. For example, it is no longer appropriate to consider having affected family members or autonomic dysfunction as exclusion criteria.^46^,^53^ Moreover, the systemic character of the disease needs to be acknowledged, especially incorporating nonmotor symptoms.^54^
3. Changes in health care or disease patterns: Exclusion criteria for patients taking neuroleptic medications^45^ require rewording, given development of atypical neuroleptics such as clozapine and quetiapine. Similarly, excluding patients with encephalitis lethargica^47^,^55^ may not be relevant as this epidemic passes into history.
4. Changes in definition that change diagnosis: For example, if early dementia is permitted, exclusion criteria targeted at cognitive impairment would be removed.

How then might future diagnostic criteria look? In developing criteria, some key aspects must be considered:

1. Clear definition of the motor syndrome: If the motor syndrome remains central to diagnosis, parkinsonism itself must be clearly defined. Several large-scale studies have documented mild parkinsonian syndromes in 25% to 50% of elderly persons without clinical features of PD^56^–^59^ or α-Syn deposition^57^,^60^; these must be distinguished from PD. Moreover, thresholds must be delineated to define parkinsonism “conversion” from prodromal stages.
2. Clinical expert as benchmark: 75% to 95% of patients diagnosed by experts during life have PD confirmed on autopsy, and more recent studies usually find better accuracy.^55^,^61^,^62^ Of note, experienced clinicians may diagnose PD with even greater accuracy than formal diagnostic criteria, perhaps because clinicians interpret exclusion criteria in context.^61^,^63^ If clinical expert opinion is the gold-standard diagnostic technique in life, then diagnostic criteria should attempt to codify the diagnostic process of an expert clinician. Criteria should systematize this process, so that it can be reproducible between clinicians (essential in research studies) and applied by clinicians with less expertise.
3. Diversity of criteria types: Imitating the diagnostic process of an experienced clinician involves incorporating numerous factors. First, clinicians assess both negative features that argue against PD and positive features that argue for PD. Second, not all features are of equal weight. Some negative features are so specific that they are incompatible with PD. Other features suggest a possible alternate cause, but are insufficient by themselves to preclude diagnosis. Third, rather than following a “recipe” to make diagnoses, experienced clinicians allow for flexibility in application of exclusion criteria, particularly if there are clear extenuating circumstances or complex situations that alter the significance of a criterion.
4. Time: Diagnostic accuracy increases with time; early in disease progression, response to treatment is less defined, and hallmarks of other neurodegenerative diseases may not have emerged. Also, some “atypical” features are incompatible with diagnosis early in disease, but may be a late feature in otherwise typical PD.
5. Grades of certainty: Overemphasis on avoiding false-positive diagnoses means missing many patients with true disease, whereas overemphasis on detecting all PD patients leads to false-positive diagnoses. The importance of false negatives versus false positives varies according to the purpose for which criteria are applied. For example, in a randomized, clinical trial, ensuring high specificity of PD diagnosis is key, and incorrect exclusion of true PD cases is arguably less critical (although excessive exclusion creates generalizability bias). By contrast, for natural history studies and epidemiologic research, diagnostic specificity and generalizability are both critical; one must balance false positives and false negatives.
6. Ancillary testing: Currently, PD diagnoses are generally made clinically, without requiring additional diagnostic tests. Any criteria should be applicable across a broad range of settings, and demanding specific ancillary diagnostic testing reduces generalizability and utility of the criteria. However, in certain contexts, ancillary diagnostic testing is used, particularly in resolving uncertain cases. Moreover, as knowledge advances, diagnostic biochemical markers, neuroimaging, or means to document α-Syn deposition may become clinically available.

Therefore ***the task force proposes*** the following:

1. Formal MDS diagnostic criteria should be created for the diagnosis of clinical PD.
2. Parkinsonism should remain a core feature of PD, based upon a combination of cardinal manifestations. The criteria should include clear definitions of what constitutes each cardinal manifestation, including explicit instructions for examination.
3. The benchmark of diagnostic criteria should be the expert clinical examination.
4. Criteria should incorporate both negative features (that argue against diagnosis) and positive features (that argue for diagnosis).
5. Criteria should be weighted, so that features that are highly specific for alternate conditions are differentiated from less specific “red flags.”
6. Criteria should incorporate a time component, such that certainty can increase with longer disease duration, and individual diagnostic criteria can be applied differentially in early versus late disease.
7. Criteria should incorporate different levels of certainty, delineated as “clinical PD” (highly specific, but not necessarily sensitive or representative) and “possible PD” (balancing specificity and sensitivity).
8. Although ancillary diagnostic tests can be incorporated, only tests that have been extensively proven as specific diagnostic markers in PD should be included. Moreover, they should be considered as ancillary only and not be essential to making diagnosis.

## Conclusion

In summary, advances in the field of PD have strained our current definition of PD. This article is meant to introduce the issue of PD redefinition and provide preliminary proposals for how to advance. Before the MDS task force creates formal diagnostic criteria, we would like to enlist the input of our members. To be part of the process, please visit the MDS website and offer your ideas and feedback.
